# Healing effects of autologous platelet gel and growth factors on cutaneous leishmaniasis wounds in addition to antimony; a self-controlled clinical trial with randomized lesion assignment

**DOI:** 10.1186/s13104-023-06470-4

**Published:** 2023-09-09

**Authors:** Enayatollah Shadmand, Kavous Solhjoo, Ali Taghipour, Akbar Hashemi Tayer, Fatemeh Sadeghi, Ahmad Meshkin

**Affiliations:** 1 Zoonoses Research Center, Jahrom University of Medical Sciences, Jahrom, Iran; 2 Department of Medical Parasitology and Mycology, School of Medicine, Jahrom University of Medical Sciences, Jahrom, Iran; 3 Research Center for Noncommunicable Diseases, Jahrom University of Medical Sciences, Jahrom, Iran; 4 School of Medicine, Jahrom University of Medical Sciences, Jahrom, Iran; 5 School of Medicine, Gerash University of Medical Sciences, Gerash, Iran

**Keywords:** Platelets gel, Cutaneous leishmaniasis, Growth factors, Wound healing

## Abstract

**Objective:**

Autologous platelet gel (APG) is used in most surgeries to treat a variety of tissue defects because of its healing factors composition. This randomized parallel clinical trial was conducted to investigate the healing effects of APG on cutaneous leishmaniasis (CL) wounds. Eighteen male patients with CL wounds were recruited and followed for two months. The patients had more than one cutaneous wound, one of which was examined as the control and the other one as the intervention wound. APG was applied to the intervention wounds once a week, up to eight times. The primary endpoint was wound healing which defined as complete epithelialization and tissue granulation. Other clinical evaluation criteria were assessment of the wound size, and histopathology analyses.

**Results:**

Of 18 patients, 15 patients completed the trial (83.3%, mean age 28 years). The use of APG on the wounds was associated with complete and faster healing in 66% of the wounds and partial healing in 34% of the wounds. During the study, none of the control wounds were completely healed. The wound area in the intervention cases showed a statistically significant decrease throughout the study (P < 0.01) compared with controls. Following treatment of CL lesions with APG, the inflammatory process in the epidermis and dermis were decreased significantly (P < 0.01) compared with controls.

**Conclusion:**

Our preliminary results confirm the clinical healing improvement described in the literature for APG-GF treatment of chronic non-leishmania wounds via immunomodulation.

*Trial registration*: IRCT, IRCT20190212042694N1. Registered 20 February 2019, https://en.irct.ir/trial/37522

## Introduction

Leishmaniasis is a protozoan disease transmitted through the bites of female sandflies [1]. Iran is one of the most popular foci of cutaneous leishmaniasis (CL), with the provinces of Ilam, Fars, Khorasan Razavi, and Isfahan having the highest prevalence rates, respectively [2]. The organisms causing leishmaniasis in Iran are *Leishmania major* (*L. major*) and *Leishmania tropica* (*L. tropica)*, with the former being the cause of most disease cases in this country. *L. major* causes rural CL (wet type) and *L. tropica* causes urban CL (dry type) [3]. Due to the long course of leishmaniasis (3–6 months in the urban type and 6–9 months in the rural type), it is also known as *Salak* in Persian language, describing a disease that lasts for about a year. CL is a self-limiting disease that, once cured, leaves scars that overshadow the patient’s beauty forever. Wounds from both types of leishmaniasis may also develop secondary infections, which prolong treatment even further [3, 4].

At present, there are physical methods (cryotherapy, laser therapy, thermotherapy), topical drugs (injection of Glucantime, Emetine hydrochloride, and application of Paromomycin ointment), and systemic drugs (Pentavalent antimony (SbV) compounds of Meglumine antimoniate and Sodium stibogluconate), which are either used alone or in combination with each other for the treatment of CL. In addition to the side effects of the mentioned drugs and their invasive injection, scarring is one of the problems that affect the patients’ physical appearance after CL [5].

Platelet gel, a blood product used for wound healing, was first developed as a byproduct of platelet rich plasma (PRP) [6]. Platelet gel is a biological product derived from human plasma that is composed of three main components: platelet-rich plasma, thrombin, and calcium. After adding thrombin and calcium to platelet-rich plasma, a thick sticky gel is formed that temporarily provides a high level of growth factors (GFs) involved in the early stages of wound healing compared to the physiological process of healing. The most important of these factors include Platelet-Derived Growth Factor (PDGF), Transforming Growth factor (TGFβ), Vascular Endothelial Growth Factor (VEGF), Epidermal Growth Factor (EGF), Fibroblast Growth Factor (FGF), and Insulin-like Growth Factor (IGF), which synergistically improve fibroblast proliferation, angiogenesis, extracellular matrix synthesis, and ultimately wound healing [7, 8]. The differential profile of growth factor expression including the PDGF seems to be of importance during the leishmania infection and response to treatment, as first described in 2018 in the CL hamster model [9]. In addition to growth factors, platelet gel contains white blood cells and biologically active substances such as serotonin, catecholamines, and antimicrobial proteins, and there is a hypothesis that platelet gel can be effective in eliminating microbial pathogens [10, 11]. In this clinical trial, we prepared a sterile APG-GFs and evaluated its effects on wound healing and scar removal in patients with CL. The present study is the first research that examines the use of APG-GFs for treatment of CL legions.

## Materials and methods

### Ethical standards

The protocol was approved by the *Jahrom university of Medical Sciences Ethic Committee* and conducted in compliance with the Declaration of Helsinki. Before enrolling patients in this study, all of them signed a written consent form after being informed about the aim, methods, benefits and any possible risk of the participation in the study. The present study was registered at the Iranian Registry of Clinical Trials and approved with the license number IRCT20190212042694N1.

### Study design and participants

This randomized, single-center, controlled, parallel clinical trial was carried out from February 2019 to January 2020. There were no important changes to methods after trial commencement.

Eighteen eligible patients with confirmed CL were enrolled in this study, out of which 15 completed the trial. The study population consisted of adult male patients with CL referred to Ghafouri Specialized Laboratory in Jahrom, Iran.

In the present study, all enrolled patients had more than one skin wound that involved the upper and lower limbs. Going for wounds of the same size and on the same limb, one of the wounds was randomly selected as the control and the other as the intervention wound.

Age older than 15 years, and the presence of at least 2 wounds with the same dimensions on the hands and feet of the patients were considered as inclusion criteria. Also, hemoglobin < 10 mg/dl, qualitative and quantitative platelet disorders, abnormal screening tests, positive history of diabetes, positive history of severe hypovolemic conditions, sepsis, and anti-inflammation medications consumption during the past 2 weeks were defined as the exclusion criteria.

**Fig. 1:**
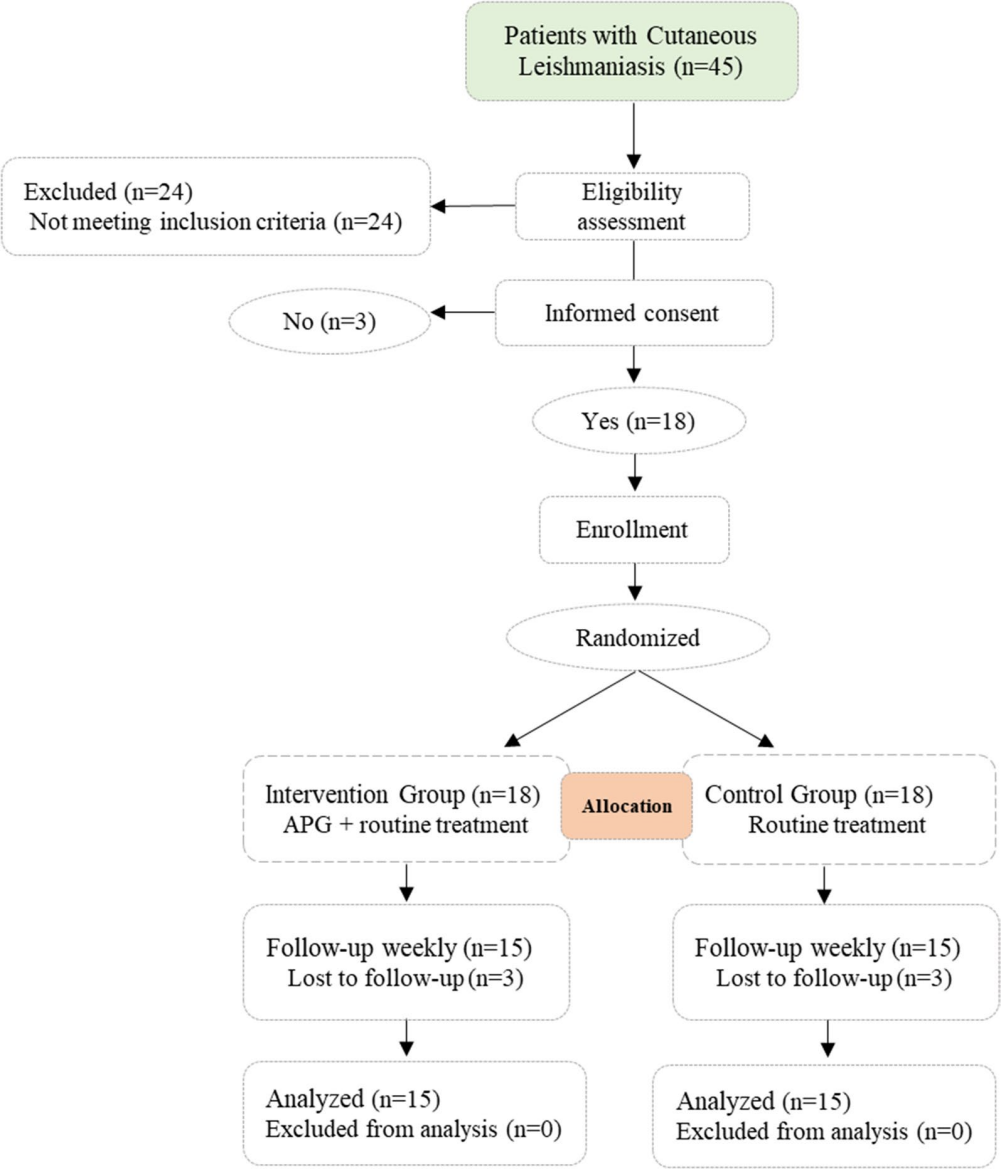
CONSORT diagram showing flow of participants throughout the study

After obtaining written consent and confirming that the prothrombin time (PT) and partial thromboplastin time (PTT) coagulation screening tests were within the normal range, 15 ml of citrated blood samples (once per week, up to 8 weeks) were taken from the patients. The recruitment and follow up flow of the participants is shown in a Consolidated Standards of Reporting Trials (CONSORT) diagram in Fig. 1 [12].

### Randomization and allocation

Randomization was done by one of the academic expert staff (not involved in this research) using randomly permuted blocks method (block size was two). Enrollment and assignment of participants to interventions was performed by the principal researcher (A.H.T) at *Jahrom University of Medical Science*. Allocation was concealed using sequentially numbered, opaque, sealed envelopes, which were opened only after the trial completion. Patients and involved personnel were not blinded to the study. Blinding was applied for outcomes investigator and pathologist.

### Parasitological studies

In this study, the smears were taken for diagnosis of CL. Skin lesions were cleaned with antiseptic agent (70% alcohol), and then using a sterile lancet, 2–3 mm superficial incisions were made on the edges of the lesions. Two smears of dermal tissue scrapings were prepared from each patient, then air-dried, fixed in methanol, and giemsa-stained. Each smear was completely scanned for Leishman body. Samples were then screened via nested PCR. Using a sterile surgical blade, the dry smear was scraped from slide, and inserted into micro tube containing 200 μl of lysis buffer (50 mg ethylene diamine tetra-acetic acid (EDTA) pH 7.4, 50 mM NaCl, 200 μg proteinase K/ml, and 1% Triton X-100). The mixture was incubated at 56 °C for 3 h. The lysate was extracted with phenol–chloroform, and finally re-suspended in 50 µl double-distilled water and kept at − 20 °C until used. Characterization of the CL isolates was done using nested PCR by specific primers of CSB1XR, and CSB2XF as external primers, and also LiR, and 13Z as internal primers, which can specifically differentiate CL species [13].

### APG-GFs preparation

To prepare the APG, platelet-rich plasma was first prepared. For this purpose, blood sample was centrifuged at 1200 rpm for 10 min and the supernatant plasma was transferred to another tube with a buffy coat and centrifuged again at 3000 rpm for 10 min. Then, to concentrate the platelets, half of the supernatant plasma was discarded, and the platelet sediment formed was kept floating in the remaining plasma and the platelet-rich plasma was thus prepared. To prepare thrombin, a reagent was made from a mixture of 25 mM calcium chloride and Merck ethanol at a 1:1 ratio. In the next step, the platelet-free plasma was mixed with thrombin reagent (at a ratio of 3:5) in a tube containing 0.5 g of glass powder and stored horizontally at room temperature for 20 min. The tube content was then mixed, and after a re-centrifugation for 10 min at 3000 rpm, the supernatant was used as the thrombin solution.

**Fig. 2:**
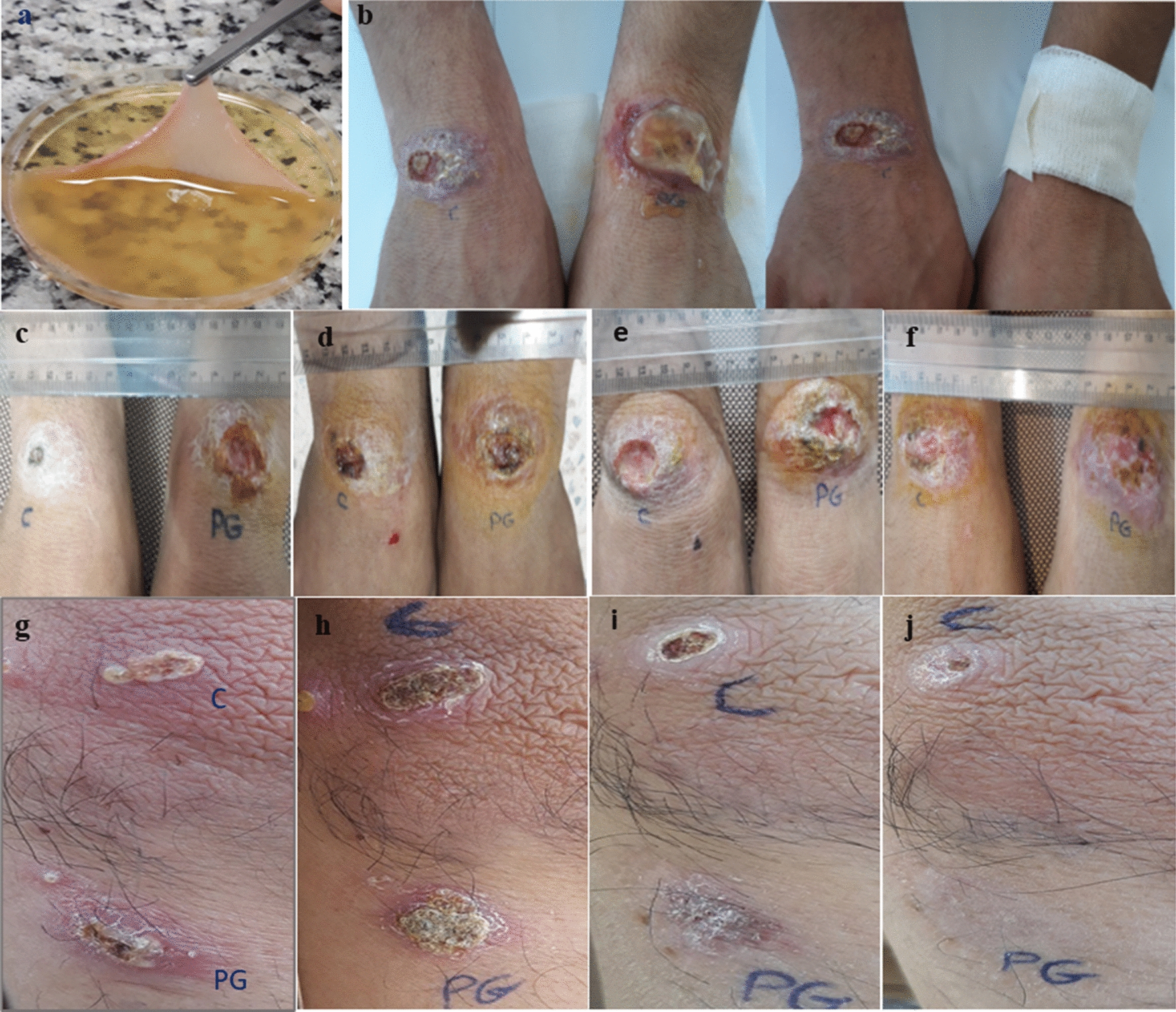
**a** Autologous platelet gel. **b** APG dressing on the wrist in patient with CL wounds. **c**–**j** CL wounds in the affected patients (**C**; Control, treated with Glucantime only, **PG**; Treated wound with APG + Glucantime): **c**, **d**, **e** and f are respectively related to CL wounds located on the wrist in 25 years old male in the first, third, sixth and 8 weeks after treatment. Follow-up revealed tissue granulation and epithelialization. **g**, **h**, **i** and **j** are respectively related to Lesions on the elbow in 31 years old man in the first, third, sixth and 8 weeks after treatment. Follow-up revealed complete recovery and scar removal

Finally, to prepare the APG, platelet-rich plasma was mixed with thrombin and calcium gluconate (at a ratio of 3;1;1.5) and incubated for 60 min at 37 °C. Following the addition of thrombin and calcium to the patients’ platelet-rich plasma, a thick, and sticky APG was formed (Fig. 2a). After the incubation time, the sample was centrifuged at 3000 rpm for 10 min and the supernatant containing the GFs was isolated [14].

### Leishmaniasis wound management

The control wound received routine treatment, including systemic injection of glucantime in a dose of 20 mg/kg/body weight, intramuscularly, daily for 14 days, and the intervention wound received APG-GF product in addition to the routine treatment. APG-GFs was prepared freshly, and applied to the wound site once per week, up to eight weeks. Before applying the gel, the supernatant containing GFs was injected into the inflamed and active area of the wound site using an insulin syringe. In addition, the wound site received approximately 3 ml of APG and was then covered and fixed with sterile gauze dressing (Fig. 2b).

### Follow-up and evaluation of the outcome

In this study, patients with CL wounds were evaluated every week for two months at the outpatient clinic by the principal investigator. The investigator was blinded to the type of treatment given to each group. Clinical evaluation of the wounds included assessment of the wound size, wound tissue granulation, epithelialization, and wound healing. The primary endpoint was complete wound healing characterized by 100% epithelialization. Reduction in wound size, and wound complications were considered as the secondary endpoints. In both groups, images were taken of the wound site before and after healing, and the dimensions of the wounds were measured using a Vernier caliper. The patients’ wounds were also examined clinically for secondary infections. Clinical criteria that were considered for local infection include erythema, heat, pus, induration, swelling, colour change, smell of exudate, and delayed wound healing.

### Biopsy collection and histopathological analysis

Biopsy samples from CL lesions were evaluated by expert pathologist before (day 0) and 8 weeks after intervention. The skin biopsy specimens were taken from the edge of cutaneous lesions with a 4 mm in thickness Harris punch, preceded by the application of local anesthesia and anti-septic agent. All specimens were fixed in 10% buffered formalin, and processed within a period of not more than 48 h to dispose of the paraffin tissue block. All specimens were dehydrated, cleared, embedded in paraffin, cut into 4 μm thick sections and stained with Hematoxylin–Eosin (HE). The histopathological changes in the epidermis (acanthosis, dyskeratosis, spongiosis, exocytosis, hyperkeratosis, and ulcer) before and after the last intervention was characterized using an optical microscope at 400 × magnification. At the same time, the intensity and distribution of inflammatory reactions in the dermis were evaluated.

### Sample size estimation

The study was designed to recruit 18 patients per treatment group. Based on previous studies and considering the following formula as the sample size equation, effect size = 0.8, α = 5%, β = 20%, the sample size was estimated at 15 patients. The sample size was increased from 15 to 18 patients to accommodate drop-outs.

$$ \begin{gathered} n = 2\left( {1.96 + 0.84} \right)^{2} \left( {\frac{1}{0.80}} \right)^{2} = 15 \hfill \\ \hfill \\ \end{gathered} $$

### Statistical analysis

Data analysis was conducted using SPSS software (version 22.0 SPSS, Inc., Chicago, IL, USA). Control and intervention cases were matched based on the confounding variables (e.g. wound size). Data were described as mean ± standard deviation (SD). Welch test was used for comparing between groups. The Kaplan–Meier method was used for the analysis of time to healing. To compare the two survival curves, the log-rank test was used. Graphs were depicted by GraphPad Prism (version 8.3). *P* value less than 0.05 was considered statistically significant.

## Results

### Patients’ characteristics

In this study, out of 45 potential participants, eighteen patients were enrolled according to inclusion criteria, thereafter, 3 patients were lost to follow up due to personal reasons, and only 15 patients completed the treatment and enrolled in the statistical analysis. Patient recruitment started in *February* 2019 and ended in *December* 2019. A CONSORT diagram is presented in Fig. 1 detailing flow of participants through the study [12].

**Fig. 3:**
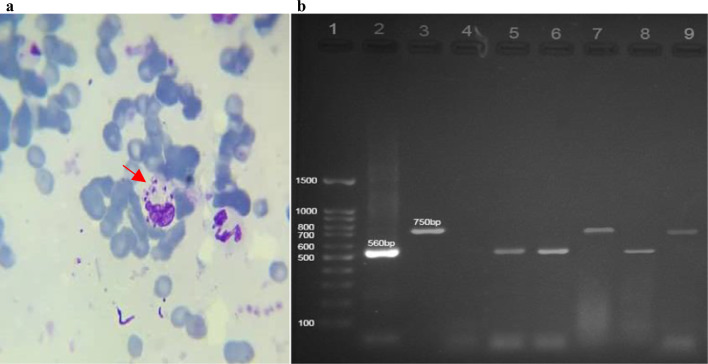
**a** Amastigotes forms of Leishmania inside macrophages in the dermis of patients affected by cutaneous leishmaniasis (red arrows). **b** Agarose gel of the PCR products of *Leishmania* species in patients with CL. Lane 1: 100 bp ladder; lanes 2 and 3: positive controls of *L. major* (560 bp), and *L. tropica* (750 bp), respectively; lane 4: negative control; lanes 5, 6 and 8: *L. major* (560 bp); lanes 7 and 9: *L. tropica* (750 bp)

The mean age of the patients was 28 ± 6.6 years. Their minimum age was 17 and maximum 38 years. All the patients were male. The results of the PT and PTT screening tests were within the normal range. The mean hemoglobin, hematocrit, and platelet count in the peripheral blood samples taken from the patients were 16.3 ± 0.86 g/dl, 49.4 ± 2.8%, and 269 ± 81.3 × 10^3^ per microliter of blood, respectively. The number of lesions ranged from 2 to 6 with an average of 3 lesions per patient, mostly located in the upper limbs (11 cases, 73.3%). Evolution time of lesions varied from 15 to 100 days with an average of 70.6 ± 11.5 days. In this study, infected CL patients verified by microscopy (Fig. 3a). In molecular study, all enrolled patients were positive for *Leishmania* spp (Fig. 3b). Of the 18 patients who were examined, 12 out of 18 (66.6%) were positive for *L. major*, and six of them were positive for *L*. *tropica* (33.3%).

### Outcome of treatment

**Fig. 4:**
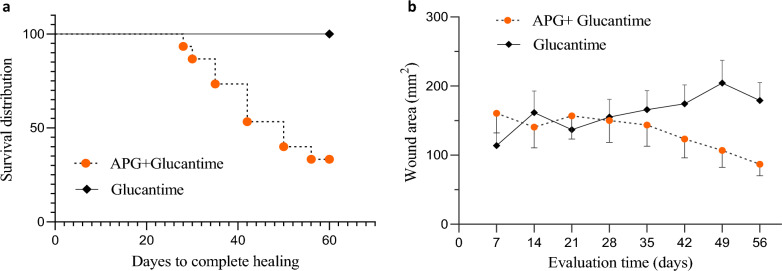
**a** The Kaplan–Meier survival plots showing patients treated with APG + Glucantime and Glucantime only, over 60 days of follow up. **b** The mean reduction in wound area and induration caused by CL following treatment: the mean wound area in the intervention group (APG + Glucantime) showed a statistically significant decrease during 8 weeks of evaluation (mean ± SEM, p < 0.01). In the control group (Glucantime only), there was no statistically significant change in wound area over time

The results showed that following the use of APG on the intervention wounds, 66% [10 cases] had complete and faster wound healing and 34% (5 cases) had partial healing. None of the control wounds were completely healed, and just 60% of them (9 cases) had partial healing during the two months of evaluation (Fig. 2c–j). Complete improvement was based on two criteria in this study; tissue granulation and epithelialization. Wounds that did not meet these criteria were regarded as cases of partial healing. According to the Kaplan–Meier analysis (Fig. 4a), the time to complete healing was shorter for the intervention wounds than for those in the control wounds (P = 0.0002, by the log-rank test). Kaplan–Meier median estimates for complete wound healing were 50 days (95% CI 3.5 to 46.7) for intervention wounds and undefined for control. The mean wound area in the intervention group showed a statistically significant decrease during the study, changing from 160.6 ± 148.9 mm^2^ in the 1 week to 86.9 ± 121.6 mm^2^ in the eighth week (Welch test, P < 0.01). The reduction in wound area in the 8 week was statistically more significant than the other times (Welch test, P < 0.01). As shown in Fig. 4b, in the control cases (treated with glucantime only), the wound area was 113.9 ± 71.2 mm^2^ in the 1 week and reached 179 ± 197.5 mm^2^ in the 8 week, but this change was not statistically significant (P = 0.06). Evaluation of the wounds for secondary infections showed that two patients met the criteria for wound infection and broad-spectrum antibiotics were prescribed for them.

### Histopathological finding

**Fig. 5:**
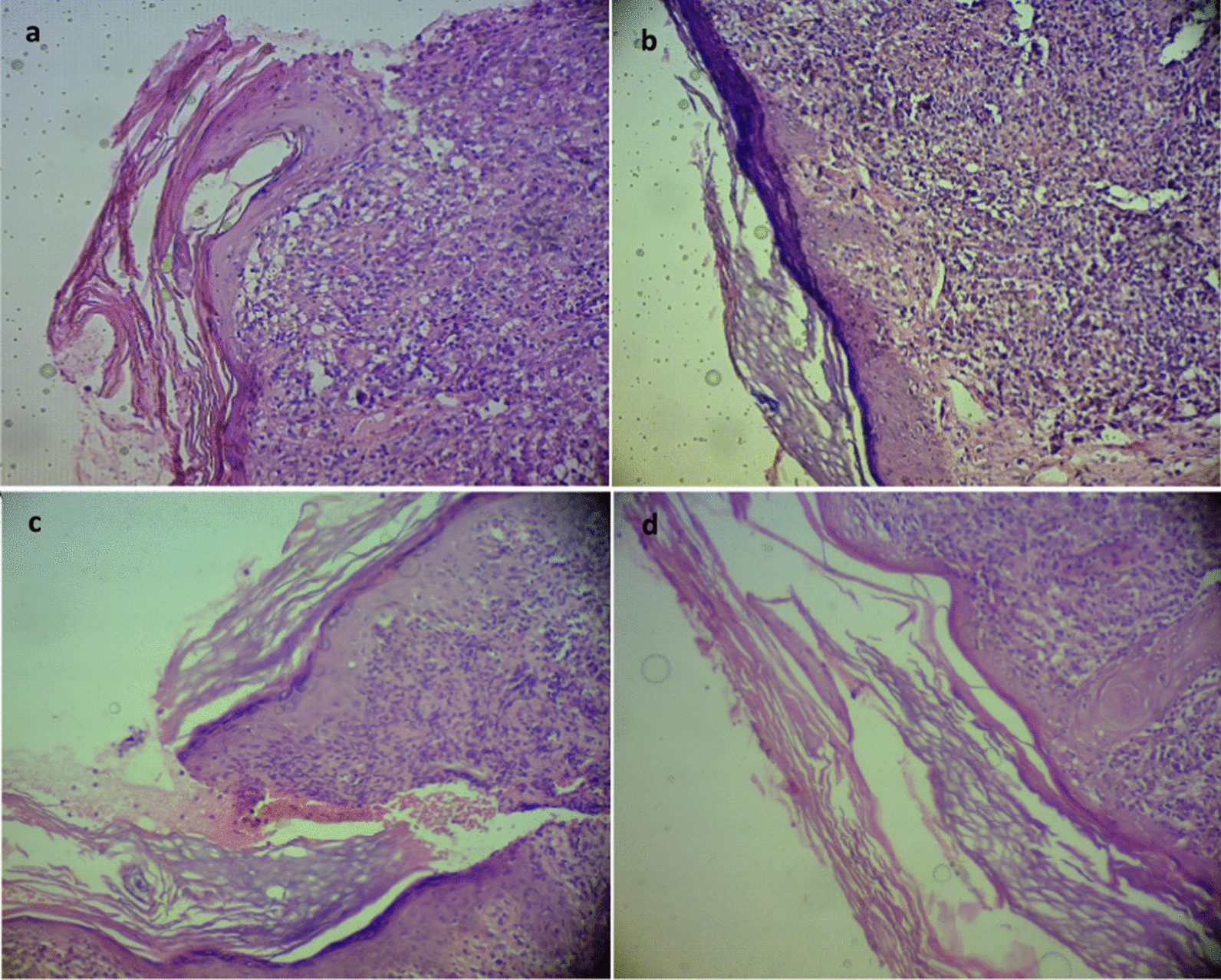
Histopathological findings observed in the CL lesions in control and intervention groups. **a** Before treatment with Glucantime only. **b** After eight weeks treatment with Glucantime only. **c** Before treatment with APG + Glucantime. **d** After 8 weeks treatment with APG + Glucantime

The histopathological analysis of the 15 skin biopsies of patients with CL showed morphological alterations in both epidermis and dermis before treatment. In treated wounds with APG and glucantime, alterations of the epidermis were observed in 80% (11/15) of skin biopsies; and were mainly characterized by acanthosis in 86.7% (13/15), spongiosis in 33.3% (5/15), hyperkeratosis in 73.3% (11/15), dyskeratosis in 40.0 (6/15), exocytosis in 26.7% (4/15), and ulcer in 46.6% (7/15) of skin biopsies. The histopathological alterations in the dermis were determined mainly by a lymphohistocytic pattern of variable intensity, with diffuse or focal distribution. The inflammatory pattern was intense in 40% (6/15), moderate in 53.3% (8/15) and light in 6.7% (1/15) of cases with diffuse distribution in 40% (6/15), and focal in 60% (9/15). The inflammatory infiltrate was characterized by predominance of lymphocytes (61%), followed by inflammatory cells. After 8 weeks of treatment with APG and glucantime, the inflammatory cells infiltrate patterns in the epidermis and dermis were decreased significantly. In treated wounds with glucantime only, a decrease in the histopathological features of the epidermis and dermis were also observed. The pattern of decrease was less significant, and some events continued in patients after the therapies (Tables1, 2, Figures5).

**Table 1:** Analysis of histopathological changes in epidermis before and after treatment

Epidermis						
	Acanthosis	Exocytosis	Spongiosis	Hyperkeratosis	Dyskeratosis	Ulcer
APG + glucantime						
Before; n (%)	13 (86.7)	4 (26.7)	5 (33.3)	11 (73.3)	6 (40.0)	7 (46.6)
After; n (%)	8 (53.3)	1 (06.6)	2 (13.3)	7 (46.6)	2 (13.3)	1 (06.6)
p-value	0.001	0.01	0.03	0.02	0.013	0.001
Glucantime						
Before; n (%)	12 (80.0)	5 (33.3)	6 (40.0)	10 (66.6)	8 (53.3)	6 (40.0)
After; n (%)	8 (53.3)	3 (20.0)	4 (26.7)	9 (60.0)	5 (33.3)	3 (20.0)
p-value	0.02	0.06	0.05	0.15	0.04	0.03

**Table 2:** Dermal histopathological characteristics before and after treatment

Dermis												
	Intensity		Distribution			Cellular type						
	Intense	Moderate	Light	Focal	Diffuse	Neutrophil		Eosinophil		Mono nuclear cells		
						≤ 10%	Absence	≤ 10%	Absence	≥ 10%	≤ 10%	Absence
APG + Glucantime												
Before; n (%)	6 (40.0)	8 (53.3)	1 (06.7)	9 (60.0)	6 (40.0)	6 (40.0)	9 (60.0)	3 (20.0)	12 (80.0)	11 (73.3)	4 (26.7)	0
After; n (%)	0	6(40.0)	9 (60.0)	11 (73.3)	4 (26.7)	2 (13.3)	13 (86.7)	1 (06.7)	14 (93.3)	0	15 (100)	0
Glucantime												
Before; n (%)	5 (33.3)	10 (66.6)	0	7 (46.6)	8 (53.3)	7 (46.6)	8 (53.3)	2 (13.3)	13 (86.6)	10 (66.6)	5 (33.3)	0
After; n (%)	0	9(60.0)	6 (40.0)	9 (60.0)	6 (40.0)	4 (26.7)	11 (73.3)	0	15 (100)	6 (40.0)	9 (60.0)	0

## Discussion

Partial or poor response of CL to standard pentavalent antimony (SbV) treatment caused by host, parasite factors [15], and as well as resistance to antimony drugs [16] is increasing.

We have therefore looked for additional healing effects of a topical treatment of CL wounds in addition to systemic SbV treatment with APG and its plasma secreted growth factors (APG-GF) which has become increasingly popular to improve healing of chronic non-leishmania wounds during the last decade. This study is the first report on the use of APG-GF for CL wound healing. We evaluated the pre- and post-treatment pathohistology of the CL lesions to find hints on a shorter and hence more cosmetic healing.

Our preliminary findings suggest that in patients with 2 CL lesions treated with 20 mg /kg body weight, SbV daily for 14 days, additionally receiving topical APG-GF for one lesion once weekly for 8 weeks improves clinical healing of this lesion in comparison to the lesion left without topical treatment.

10 (66%) of the APG-GF treated wounds healed completely till day 56, and 5 (34%) lesions showed partial healing. The Kaplan–Meier wound of both lesion groups confirm faster healing of the APG-GF treated CL lesions. Meanwhile, no statistically significant change was observed in the wound healing, as well as wound area over time in the control group.

Martins et al. study showed that the use of platelet gel can be highly effective in the treatment of various types of chronic wounds [17]. In addition, Elgarhy et al., reported that topical platelet gel is an effective, low cost, and safe procedure in enhancing the healing of chronic ulcers [7].

Researches on wound healing have shown that platelet gels accelerate wound healing, epithelialization, and angiogenesis. Platelets induce an intense inflammatory response in the used site and play a key role in the healing of the injured tissue [18].

Gupta et al., reported that the inflammatory response and tissue repair of burn lesions treated with autologous platelet concentrate were significantly different compared to the control group. In their study, all patients who received the intervention demonstrated significantly better graft uptake rate when compared to patients in the control group [19].

In another study, Elsaid et al. showed that platelet gel dressing is clinically effective in healing diabetic wounds and use of platelet gel dressing for diabetic foot ulcer resulted in a more significant reduction in the size of the ulcer when compared to routine dressing.

Also, the time to reach the point of maximal healing with the least wound dimensions was significantly shorter when using platelet gel [20]. Henderson et al., found that the use of each of platelet growth factors generated marginal acceleration of re-epithelialization [21]. A comprehensive review study also suggested that the clinical use of GFs was effective in the healing of diabetic wounds [22].

APG is rich in growth factors and its application on the injury site summons the inflammatory cells and causes the onset of cell differentiation, which in turn accelerates the wound healing process and can be used as an auxiliary treatment in wound healing [23]. Platelet gel can be prepared both autologously and homologously. The advantages of APG products include safety, inexpensiveness, availability, non-transmission of viral diseases, and causing no immunological reactions [24, 25].

The results of the present study showed that the use of APG accelerates the formation of granulation tissue. An increase in granulation tissue and accelerated wound healing was observed in the patients who used APG, which is consistent with the results of similar studies on this subject [6, 20]. Platelet gel increases tissue vessels via angiogenesis and tissue granulation via effective collagen synthesis. Platelet gel also has biologically active substances and antibacterial proteins that appear to play a role in the accumulation of white blood cells and thus has an antimicrobial activity [10].

In the present study, histopathological findings of CL lesions were also represented.

Botelho et al. found that evaluation of histopathological samples is important for analyzing and understanding of the tissue healing process [26]. The presence of inflammatory cells with predominance of lymphocytes was observed in the CL lesions before treatment, whilst the biopsies from 8 weeks APG treated lesions were determined by the presence of a moderate to mild inflammation. After 8 weeks treatment of CL lesions with APG, the inflammatory patterns in the epidermis, and dermis were decreased significantly, indicating a histopathological healing process. The histopathological cure could be indicated by the absence of an inflammatory cells infiltrate [26].

Although, in some cases even after complete treatment of patients, when the CL lesions were considered clinically healed, an inflammatory pattern still persisted in lesions. Similar histopathological features have been described in Viana, et al. reports [27] which analyzed biopsies obtained from patients infected with American CL. Their findings showed that the histopathological test did not always correlate with the indications of a clinical cure [27]. In some studies, biopsies were performed on the treated patients with CL scars where the presence of inflammatory cells was observed; some patients even presented a mild inflammatory pattern after 3 years of complete healing [28, 29].

Generally, the results of present study showed that wounds caused by CL may heal faster and more effectively with the additional use of APG than the standard anti-protozoal chemotherapy with SbV alone. The presence of moderate to intense infiltrates of inflammatory cells noticed before APG-GF treatment start was not absent after the end of APG-GF treatment, even with a complete clinical cure, the subjects still showed a mild inflammatory process.

## Limitations

One of the limitations of this study was that we could not use APG continuously due to the short life span of platelets at room temperature, and for optimal healing effect, it should be prepared fresh. In addition, further trials are needed to confirm our preliminary findings by increasing the patient number and the duration of treatment and by eventually adding a second clinical design by comparing three lesions in one patient, one treated with topical SbV first and with APG-GF thereafter and the second lesion with APG-GF first, and with topical SbV only, if there is insufficient healing after 8 weeks.

## Data Availability

The datasets that analyzed during the current study is available through corresponding author on reasonable request.

## Abbreviations

APG: Autologous platelet gel
CL: Cutaneous leishmaniasis
EGF: Epidermal growth factor
FGF: Fibroblast growth factor
GFs: Growth factors
HE: Hematoxylin eosin
IGF: Insulin-like growth factor
PDGF: Platelet-derived growth factor
PRP: Platelet rich plasma
PT: Prothrombin time
PTT: Partial thromboplastin time
TGF: Transforming growth factor
VEGF: Vascular endothelial growth factor
